# Role of Barium Esophagography in Patients with Locally Advanced Esophageal Cancer: Evaluation of Response to Neoadjuvant Chemoradiotherapy

**DOI:** 10.1155/2013/502690

**Published:** 2013-12-04

**Authors:** Daisuke Tsurumaru, Kiyohisa Hiraka, Masahiro Komori, Yoshiyuki Shioyama, Masaru Morita, Hiroshi Honda

**Affiliations:** ^1^Department of Clinical Radiology, Graduate School of Medical Sciences, Kyushu University, 3-1-1 Maidashi, Higashi-ku, Fukuoka City 812-8582, Japan; ^2^Department of Heavy Particle Therapy and Radiation Oncology, Graduate School of Medical Sciences, Kyushu University, 3-1-1 Maidashi, Higashi-ku, Fukuoka City 812-8582, Japan; ^3^Department of Surgery and Sciences, Graduate School of Medical Sciences, Kyushu University, 3-1-1 Maidashi, Higashi-ku, Fukuoka City 812-8582, Japan

## Abstract

*Purpose*. This retrospective study examined the usefulness of barium esophagography, focusing on the luminal stenosis, in the response evaluation of neoadjuvant chemoradiotherapy (NACRT) in patients with esophageal cancer. *Materials and Methods*. Thirty-four patients with primary advanced esophageal cancer (≥T2) who were treated with NACRT before surgical resection were analyzed. All patients underwent barium esophagography before and after NACRT. The tumor length, volume, and percent esophageal stenosis (PES) before and after NACRT were measured. These values and their changes were compared between histopathologic responders (*n* = 22) and nonresponders (*n* = 12). *Results*. Posttreatment tumor length and PES in responders (4.5 cm ± 1.1 and 33.0% ± 18.5) were significantly smaller than those in nonresponders (5.8 cm ± 1.9 and 48.0% ± 12.9) (*P* = 0.018). Regarding posttherapeutic changes, the decrease in PES in responders (31.5% ± 13.9) was significantly greater than that in nonresponders (14.4% ± 10.7) (*P* < 0.001). The best decrease in PES cutoff with which to differentiate between responders and nonresponders was 18.8%, which yielded a sensitivity of 91% and a specificity of 75%. *Conclusions*. Decrease in PES is a good parameter to differentiate responders from nonresponders for NACRT. Barium esophagography is useful in response evaluation to NACRT in patients with locally advanced esophageal cancer.

## 1. Introduction

In the treatment evaluation of chemoradiotherapy in patients with esophageal cancer, new guidelines published in 1999, known as the “Response Evaluation Criteria in Solid Tumors (RECIST),” have been commonly used [1]. RECIST gives specific size requirements for measurable lesions at baseline to distinguish target from nontarget lesions. It is difficult to measure accurately the primary site of esophageal cancer as distinct from the normal esophageal wall in one dimension, because a computed tomography (CT) scan detects a primary lesion of esophageal cancer according to wall thickness of the esophagus. Therefore, the primary site of esophageal cancer is often identified as a “nontarget lesion” [2]. Accordingly, in the case of patient who has no target lesion (i.e., nodal involvement), evaluation of response to chemoradiotherapy is not clinically available. The only way to verify the response is to pathologically evaluate the resected specimen after the treatment, neoadjuvant chemoradiotherapy (NACRT). NACRT is a treatment option for advanced esophageal cancer which main aim is downstaging before surgery to increase rates of curative resection [3, 4].

Barium esophagography has not generally been used in evaluating the response to chemoradiotherapy, because accurate measurement of esophageal tumor using barium esophagography was also considered to be difficult due to its diverse nature [5]. However, barium esophagography has a high potential of describing esophageal lesion and is useful for diagnosing depth of invasion of esophageal cancer [6].

The purpose of this study was to clarify whether evaluation of response to chemoradiotherapy is possible, by comparing the findings of double-contrast barium esophagography with histopathologic response in patients with esophageal cancer who underwent NACRT.

## 2. Methods and Materials

This study was performed with approval of the institutional review board of our institution.

### 2.1. Patients

We retrospectively analyzed 34 consecutive patients with primary advanced esophageal cancer (≥T2) who were treated with NACRT before surgical resection during the period from July 2006 to June 2011 at our institution. Stratification to initial T2–T4 category was based on the findings of EUS, CT, and FDG-PET. All patients included in this study had histologically diagnosed squamous cell carcinoma of the esophagus and underwent barium esophagography before and after NACRT. Patients were excluded if they had a previous or secondary malignancy, or had previously undergone radiation therapy, chemotherapy, endoscopic therapy, or had nonstenotic (polypoid) type tumor. Finally, the study group comprised 30 men and 4 women, with an age range of 47–82 years (mean age 62 years). The patients' profiles are shown in Table 1.

### 2.2. Treatment

Radiotherapy was performed using external photon beams delivered at a daily dose of 1.8 Gy, five times per week, at a dose of 38–41.4 Gy (mean 41.3 Gy). The concurrent chemotherapy consisted of cisplatin (CDDP) and 5-fluorouracil (5-FU) with a dose of 5–9 mg/m^2^/d (mean 7.1 mg/m^2^/d) and 250–500 mg/m^2^/d (mean 413 mg/m^2^/d), respectively. With an interval of 3–10 weeks after the completion of NACRT, patients underwent standard right thoracic esophagectomy with modified 3-field lymphatic dissection.

### 2.3. Esophagography

**Figure 1:**
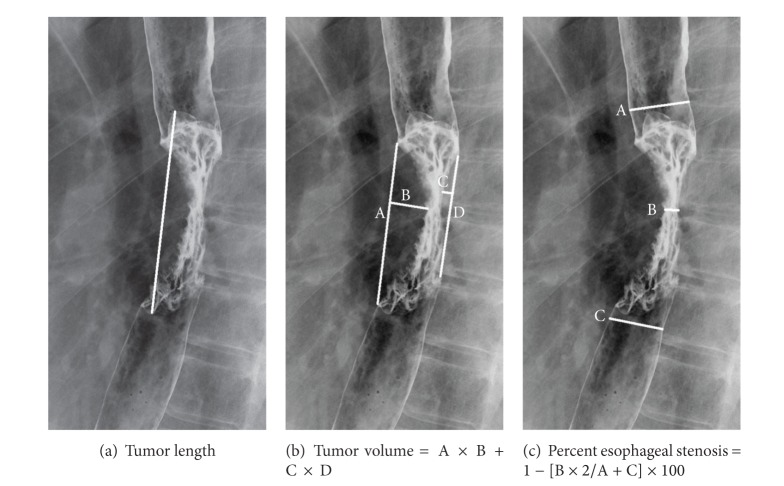
Measuring methods of esophageal stenosis.

Both initial and second barium study were performed using double-contrast esophagography technique. To produce hypotonus of the esophagus, 20 mg of butyl scopolamine (Buscopan; Boehringer Ingelheim, Tokyo, Japan) was intramuscularly injected just before examination. The double-contrast esophagography images were obtained with a 170% w/v (weight/volume) suspension of barium (Baritogen HD; Fushimi Pharmaceutical Co., Ltd., Kagawa, Japan) and gas ingested via a 12 Fr nasogastric tube. In different positions (anterior-posterior, lateral, and right/left oblique) with multiple projections, the narrowest projection of the lesion and the most distended normal esophagus were chosen to prepare calibration. Tumor volume was determined, using conventional bidimensional measurement, by multiplying the maximal measured longitudinal length and perpendicular depth of the tumor [7, 8]. The percent esophageal stenosis (PES) was based on the diameter across the lesion at maximal narrowing and the average of the normal oral and anal side diameters by the following formula: PES = [(average of normal diameters − diameter of maximal narrowing)/average of normal diameters] × 100 (Figure 1) [6]. The second esophagography, for treatment evaluation, was performed 2 to 4 weeks after the completion of NACRT.

### 2.4. Histopathologic Analysis

Histopathologic responses were determined in the primary tumor site after operation according to the guidelines of the *Clinical and Pathologic Studies on Carcinoma of the Esophagus, the Japan Esophageal Society* [8]. The grading of histopathologic response was determined as follows: grade 0 indicates ineffective, grade 1 indicates slightly effective (viable cells occupied more than one-third of the entire tumor), grade 2 indicates moderately effective (viable cells occupied less than one-third of the entire tumor), and grade 3 indicates markedly effective (absence of residual tumor). All patients who demonstrated grade 0 or 1 regression were considered to be histopathologic nonresponders. All patients who showed grade 2 or 3 regression were considered to be responders.

### 2.5. Statistical Analysis

Pretreatment, posttreatment, and decrease in tumor length, volume (conventional volumetry), and PES were compared between responders and nonresponders using Student's *t*-test. *P* values less than 0.05 were considered to be statistically significant. To determine the best cutoff value with which to differentiate responders from nonresponders, we constructed a receiver operating characteristic curve (ROC). These statistical analyses were conducted with statistical software JMP (version 8.0; SAS Institute, Cary, NC).

## 3. Results and Discussion

**Figure 2:**
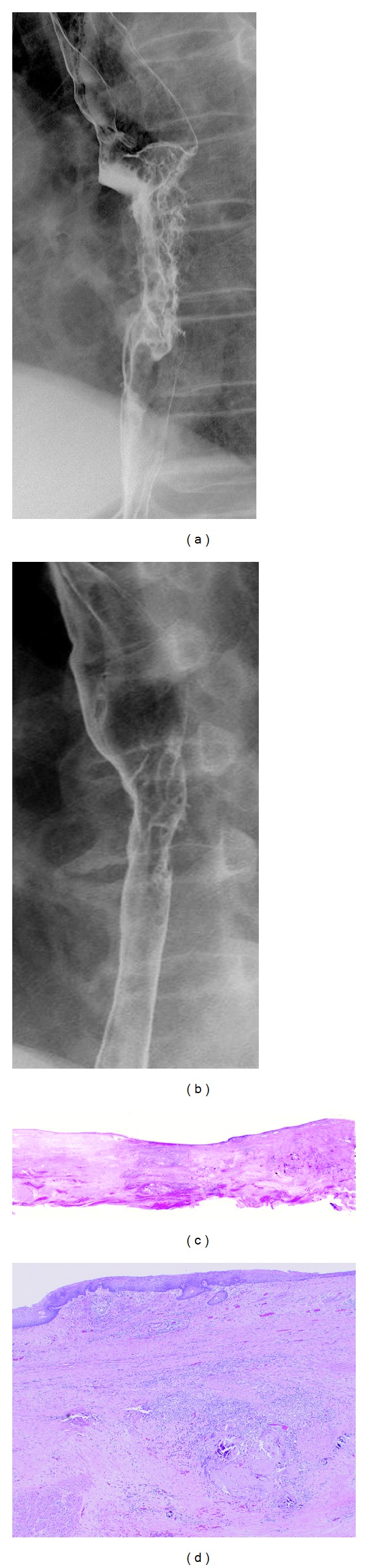
76-year-old male with esophageal cancer who obtained a grade 3 pathologic response. (a) Before NACRT, esophagography shows irregular wall stenosis in the middle esophagus with the PES of 55.6%. (b) After NACRT, esophageal wall stenosis has improved result in the PES of 33.8%, which indicates the decrease in PES of 18.8%. (c), (d) Pathological specimen of the resected esophagus shows no carcinoma cells (grade 3). Many degenerative cells with keratinization and diffuse fibrosis are seen in the submucosa and muscularis propria.

**Figure 3:**
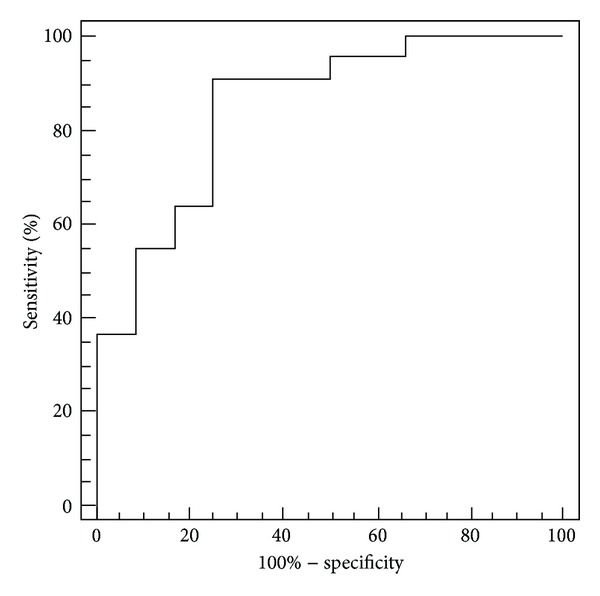
ROC analysis. The best decrease in PES cutoff with which to differentiate between responders and nonresponders is 18.8%, which yields a sensitivity of 91% and a specificity of 75%.

Histopathologic specimens showed 22 responders (grade 2 or 3) and 12 nonresponders (grade 0 or 1). There was no significant difference in pretreatment tumor length, volume, and PES between responders and nonresponders. Posttreatment tumor length and PES in responders (4.5 cm ± 1.1 and 33.0% ± 18.5) were significantly smaller than those in nonresponders (5.8 cm ± 1.9 and 48.0% ± 12.9) (*P* = 0.018). However, there was no significant difference in posttreatment tumor volume between responders (4.0 cm^2^ ± 2.8) and nonresponders (4.7 cm^2^ ± 2.7) (*P* = 0.445). Regarding posttherapeutic changes, decrease in PES in responders (31.5% ± 13.9) was significantly greater than that in nonresponders (14.4% ± 10.7) (*P* < 0.001). However, there was no significant difference in decrease in tumor length and volume between responder (14.7% ± 16.1 and 56.7% ± 24.9) and nonresponder (8.9% ± 8.9 and 44.8% ± 26.1 ) (*P* = 0.269 and 0.198) (Table 2) (Figure 2). In the ROC analysis, area under the curve of decrease in PES was 0.84, and that of posttreatment tumor length and PES were 0.69 and 0.75. The best decrease in PES cutoff with which to differentiate between responders and nonresponders was 18.8%, which yielded a sensitivity of 91% and a specificity of 75% (Figure 3).

The results of this study indicated that posttreatment tumor length and PES in responders were significantly smaller than those in nonresponders, and that decrease in PES in responders was significantly greater than that in nonresponders. The change of PES of barium esophagography might reflect the changes in tumor volume. Ito et al. reported that barium esophagography was useful diagnostic tool in the tumor staging of esophageal cancer and that the accuracy rate of the depth of invasion with barium esophagography was comparable to EUS [6]. The PES of barium esophagography increases according to the depth of tumor invasion, which is highly associated with tumor volume.

There was no significant difference in other parameters such as posttreatment tumor volume, decrease in tumor volume, and decrease in tumor length between responders and nonresponders. These results may support the inaccuracy of tumor volume measurement on 2 dimensional images such as esophagography. Regarding the posttreatment tumor length, it may not be suitable for the response evaluation, because it is very difficult to demarcate the ill-defined tumor from normal esophagus after chemoradiotherapy.

The results of this study indicated that a double-contrast barium esophagography using PES differentiated between responders and nonresponders with the sensitivity of 91% and specificity of 75%. Endoscopic ultrasound (EUS) or F-18 fluorodeoxyglucose positron emission tomography (FDG-PET) has been used for evaluation of therapeutic response in patients with esophageal cancer; the sensitivity and the specificity are 50% to 100% and 36% to 100% for EUS [9–12], and 50% to 100% and 55% to 100% for FDG-PET [10, 13–16]. Our result indicated that the diagnostic performance of barium esophagography could be comparable to EUS or FDG-PET.

In recent years, barium esophagography has not generally been used in evaluating the therapeutic response, because quantitative assessment of esophageal tumor using conventional volumetry was considered to be difficult due to its diverse nature [5]. Even pathologically markedly effective cases present esophageal wall thickening related to inflammatory change or fibrosis without residual cancer [17–19]. There were 10–11.9% mismatched cases shown to have a pathological complete response despite being diagnosed with residual tumors [20, 21]. Several studies investigated previously the use of endoscopic biopsy in predicting the pathological response to neoadjuvant therapy [22–24]. However, these studies suggested that endoscopic biopsy is not reliable for determining the presence of residual disease because of higher rates of false negative results.

In our study, most responders after NACRT had some degree of esophageal stenosis owing to inflammatory change or fibrosis. In clinical setting, it is more practical to differentiate responder from nonresponder rather than to diagnose no residual cancer, because it has been recently shown that patients responding to neoadjuvant therapy had a better survival than patients not responding to neoadjuvant therapy [25–28]. It is also useful if our results can be adapted to the response evaluation of definite chemoradiotherapy. Treatment response of definite chemoradiotherapy is generally determined by imaging examination or follow-up investigation several months later not by pathological findings. In the course of definitive chemoradiotherapy, a method that can be used to predict therapeutic response early after initiating chemoradiation is crucially important for avoiding chemoradiation-related side effects and unnecessary delay for surgery.

In the diagnosis or treatment evaluation of esophageal cancer, barium esophagography is the primary imaging technique, which is simple to perform, inexpensive, and noninvasive. Furthermore, double-contrast esophagography reveals the mucosal appearance and enables good reproduction of lesions. The value of barium esophagography should be reviewed because it can be useful for evaluation of treatment response to Chemoradiotherapy as well as staging of locally advanced esophageal cancer.

There are some limitations that need to be addressed regarding this study. First, the patients were examined between 2 and 4 weeks and operated between 3 and 10 weeks after completion of NACRT. There was great variability in the time interval between the examination and the operation among patients, which might have influence on our result. Secondly, association of nodal involvement or other prognostic factors were not discussed. They might be also important factors for evaluating the response of NACRT.

## 4. Conclusions

Decrease in PES after chemoradiotherapy is a good parameter to differentiate responders from nonresponders for NACRT. Barium esophagography, commonly or traditionally used modality, has still been a useful diagnostic tool which could determine the response to NACRT in patients with locally advanced esophageal cancer.

## Figures and Tables

**Table 1:** Patient and tumor characteristics (*n* = 34).

Mean age (range), y	62 (47–82)
Male/female no.	30/4
Pathology no.	
Squamous cell carcinoma	34
Tumor stage no.	
T2	2
T3	23
T4	9
Tumor location no.	
Ce	3
Ut	7
Mt	15
Lt	8
Ae	1
Mean ± SD total radiation dose, Gy	41.3 ± 1.8
Chemotherapy regimen no.	
CDDP + 5-FU	34

Ce: cervical esophagus; Ut: upper thoracic esophagus; Mt: middle thoracic esophagus; Lt: lower thoracic esophagus; Ae: abdominal esophagus; SD: standard deviation; CDDP: cisplatin; FU: fluorouracil.

**Table 2:** Tumor length, volume, and PES.

	Responders (*n* = 22)	Nonresponders (*n* = 12)	*P* value
Tumor length			
Pretreatment (cm)	5.5 ± 1.9	6.2 ± 2.3	0.336
Posttreatment (cm)	4.5 ± 1.1	5.8 ± 1.9	0.018
Decrease (%)	14.7 ± 16.1	8.9 ± 8.9	0.269
Tumor volume			
Pretreatment (cm^2^)	10.3 ± 7.6	8.5 ± 3.3	0.439
Posttreatment (cm^2^)	4.0 ± 2.8	4.7 ± 2.7	0.445
Decrease (%)	56.7 ± 24.9	44.8 ± 26.1	0.198
PES			
Pretreatment (%)	64.5 ± 12.9	62.4 ± 13.5	0.659
Posttreatment (%)	33.0 ± 18.5	48.0 ± 12.9	0.018
Decrease (%)	31.5 ± 13.9	14.4 ± 10.7	<0.001

Note: data are means ± standard deviations. PES: percent esophageal stenosis.
